# Comprehensive In Vitro Evaluation of Antibacterial, Antioxidant, and Computational Insights into *Blepharis ciliaris* (L.) B. L. Burtt from Hail Mountains, Saudi Arabia

**DOI:** 10.3390/plants13243491

**Published:** 2024-12-13

**Authors:** Abdel Moniem Elhadi Sulieman, Hajo Idriss, Mamdouh Alshammari, Nujud A. M. Almuzaini, Nosyba A. Ibrahim, Mahmoud Dahab, Abdulrahman Mohammed Alhudhaibi, Hamad Mohammed Abdullah Alrushud, Zakaria Ahmed Saleh, Emad M. Abdallah

**Affiliations:** 1 Department of Biology, College of Science, University of Hail, Hail 2440, Saudi Arabia; 2 Department of Physics, College of Science, Imam Mohammad Ibn Saud Islamic University (IMSIU), Riyadh 11623, Saudi Arabia; 3 Deanship of Scientific Research, Imam Mohammad Ibn Saud Islamic University (IMSIU), Riyadh 11432, Saudi Arabia; 4 Department of Public Health, College of Public Health & Health Informatics, University of Hail, Hail 2440, Saudi Arabia; nos.ahmed@uoh.edu.sa; 5 Faculty of Pharmacy, University of Malaya, Wilayah Persekutuan Kuala Lumpur 50603, Malaysia; mahmouddahab4@gmail.com; 6 Department of Biology, College of Science, Imam Mohammad Ibn Saud Islamic University (IMSIU), Riyadh 11623, Saudi Arabia; 7 Department of Research and Training, Research and Training Station, King Faisal University, Al-Ahsa 31982, Saudi Arabia; 8 Department of Biology, College of Science, Qassim University, Qassim 51452, Saudi Arabia

**Keywords:** antibacterial agents, antioxidant, in vitro study, chemical composition, computational biology

## Abstract

The arid mountainous region of Hail in Saudi Arabia has a variety of desert vegetation, some of which are conventionally used in Bedouin traditional medicine. These plants need scientific examination. This research seeks to examine *Blepharis ciliaris* using a thorough multi-analytical methodology that includes antibacterial and antioxidant assessments as well as computational modeling. GC–MS analysis of the methanolic extract revealed 17 organic compounds, including pentadecanoic acid, ethyl methyl ester (2.63%); hexadecanoic acid, methyl ester (1.00%); 9,12-octadecadienoic acid (Z,Z)-, methyl ester (2.74%); 9-octadecenoic acid, methyl ester (E) (2.78%); octadecanoic acid (5.88%); 9-tetradecenoic acid (Z) (3.22%); and undec-10-enoic acid, undec-2-n-1-yl ester (5.67%). The DPPH test evaluated antioxidant activity, revealing a notable increase with higher concentrations of the methanolic extract, achieving maximum inhibition of 81.54% at 1000 µg/mL. The methanolic extract exhibited moderate antibacterial activity, with average inhibition zones of 10.33 ± 1.53 mm, 13.33 ± 1.53 mm, 10.67 ± 1.53 mm, and 10.00 ± 2.00 mm against *Staphylococcus aureus*, *Bacillus subtilis*, *Escherichia coli*, and *Serratia marcescens*, respectively, as determined by the disk diffusion method. The minimum inhibitory concentration (MIC) values were 500 µg/mL for *S. aureus* and *B. subtilis*, whereas *E. coli* and *S. marcescens* showed susceptibility at 1000 µg/mL. Computational simulations were employed to assess the toxicity, drug-likeness, and ADMET profiles of compounds derived from *Blepharis ciliaris*. Thirteen bioactive compounds were assessed in silico against *Staphylococcus aureus* sortase A (PDB: 1T2O), *Bacillus subtilis* BsFabHb (PDB: 8VDB), *Escherichia coli* LPS assembly protein (LptD) (PDB: 4RHB), and a modeled *Serratia marcescens* outer-membrane protein TolC, focusing on cell wall and membrane structures. Compound 3, (+)-Ascorbic acid 2,6-dihexadecanoate, shown significant binding affinities to *B. subtilis* BsFabHb, *E. coli* LPS assembly protein, and *S. marcescens* TolC.

## 1. Introduction

Since antiquity, humans have relied on medicinal plants as their primary source for diverse and powerful medicines [1]. Even with the tremendous advancements in technology, particularly in the field of health, there are still chronic and pandemic illnesses that are difficult for contemporary therapies to adequately cure. By 2030, it is projected that the global expenses of chronic illnesses, such as diabetes, heart disease, and respiratory disorders, would amount to $47 trillion. Individual lifestyle decisions, routines, and social settings have a significant impact on how these disorders develop and are treated [2]. However, microbial infections have reached dangerous heights, and a number of bacteria are displaying increased antibiotic resistance, which raises the possibility of a return to the pre-antibiotic period. Since the 1980s, no new classes of antibiotics have been identified. Since then, rather than being completely novel substances, the majority of newly created antibiotics have been modifications or adaptations of already-existing classes [3,4].

Plants remain the primary source of healthcare for about 80% of the world’s population, and the trade of medicinal plants continues to be popular [5]. Globally, between 50,000 and 80,000 species of flowering plants are utilized for medicinal purposes [6]. Medicinal plants are abundant sources of diverse bioactive phytochemicals or bionutrients. These include primary phytochemicals, which are essential nutritive compounds like common sugars, amino acids, proteins, nucleic acid components such as purines and pyrimidines, and chlorophyll. Furthermore, they include secondary phytochemicals, which are bioactive compounds, such as alkaloids, terpenes, flavonoids, lignans, plant steroids, curcumins, saponins, phenolics, and glycosides [7,8]. Consequently, it is crucial to scientifically verify plant characteristics, particularly those that remain inadequately studied due to their occurrence in remote regions. This study offers essential insights into the interactions between bioactive compounds and biological systems, which are vital for elucidating therapeutic effects, mechanisms of action, and prospective medical applications.

The Arabian Peninsula is characterized by aridity and limited biodiversity, yet it supports a range of flora, including numerous culinary and medicinal plants adapted to extreme conditions. Nevertheless, the therapeutic potential of these adaptable species has not garnered substantial attention from the scientific community [9]. The investigation of medicinal plants in Saudi Arabia remains in its early phases. Approximately 300 plant species, representing merely 12% of the total flora, have been recognized for their medicinal potential. The species are distributed among 72 plant families, within the context of approximately 2250 known species in the flora of Saudi Arabia. Approximately 1950 species across 142 families have yet to be investigated for their potential medicinal uses [10]. Rapid urbanization and continuous human activities in Saudi Arabia may negatively impact the provision of ecosystem services, especially those originating from native plant species. The sustainable provision of these services by natural ecosystems can be enhanced through the adoption of land management practices that prioritize species restoration and mitigate pressures on native vegetation [11].

*Blepharis ciliaris* (L.) B.L. Burtt is a desert plant that inhabits arid regions and is indigenous to the Arabian Peninsula, southern Iran, and Pakistan [12]. *Blepharis* is a large genus within the Acanthaceae family, with more than 126 species. Species of Blepharis possess distinctive blooms with vibrant petals, and several species have economic significance for their use as natural dyes and ornamental items [13]. Several species of *Blepharis*, including *B. ciliaris*, *B. edulis*, *B. linariifolia*, *B. scindica*, and *B. maderaspatensis*, are extensively utilized in traditional medicine across various African and Asian countries for the treatment of a range of infectious and chronic diseases, inflammatory conditions, skeletal disorders, and parasitic problems [14]. In folk medicine, coughs and asthma are treated with an oral infusion of the plant’s leaves, roots, and seeds [15]. Various species of *Blepharis* have garnered the attention of researchers in scientific studies, leading to inquiries into their therapeutic properties. Numerous pharmacological actions have been shown in several species of the *Blepharis* genus, including anti-inflammatory and analgesic effects, wound-healing properties, gastroprotective abilities, as well as antioxidant, antibacterial, and anticancer activities [16]. These data highlight the significant therapeutic potential of this genus and its suitability for further pharmacological research. Nonetheless, investigations into the chemical contents and therapeutic properties of extracts from the *Blepharis* plant, as well as the isolated compounds from *Blepharis* species, are limited, especially for those cultivated in the dry regions of Saudi Arabia. Consequently, a comprehensive understanding of the scientific advancements about the genus, particularly its traditional use, phytochemistry, and pharmacological properties, is crucial for formulating future research endeavors connected to these species.

The bacterial cell envelope is a complex structure composed of cell wall and cell membrane, this structure plays a key role in viability, virulence, mechanical integrity [17], nutrient uptake, waste excretion, and signaling [18]. Gram-negative bacteria surrounding the cell wall is the outer membrane (OM). Importantly, the outer membrane of Gram-negative bacteria contains lipopolysaccharides (LPS), which have important roles for causing disease. Moreover, LPS acts as a barrier against small hydrophobic molecules and antibiotics while requiring complex transport mechanisms, such as the lipopolysaccharide transport DE (LptDE) complex, for its proper localization [19]. Sortase A (SrtA) is a protein on the surface of bacteria that acts as anchoring virulence factors to the cell surface of bacteria, facilitating their interaction with host tissues [20,21,22]. Notably, different enzymes, such as β-Ketoacyl-Acyl Carrier Protein Synthase IIIa (FabHa) and β-Ketoacyl-Acyl Carrier Protein Synthase IIIb (FabHb), have varied functions in controlling the membrane’s fatty acid composition, which are incorporated into the membrane, and β-Ketoacyl-Acyl Carrier Protein Synthase II (FabF) enzyme aids in the elongation of fatty acid chains [23]. Targeting FabH, a crucial enzyme that initiates fatty acid synthesis in bacteria, offers a viable strategy for antibiotic development, since interrupting fatty acid biosynthesis might compromise bacterial membrane integrity. FabH primarily influences lipid metabolism, whereas distinct pathways targeting cell wall and membrane-associated proteins, such as sortase A, outer membrane channel protein (TolC), and lipopolysaccharide transport protein D (LptD), provide promising avenues for innovative antibiotic methods due to their functions in bacterial construction, efflux, and outer-membrane transport [24].

Structure-based drug design (SBDD) is a technique that uses three-dimensional models of target molecules, whether from X-ray crystallography, nuclear magnetic resonance (NMR), or homology modeling, to efficiently identify small compounds that may bind to and possibly address disorders. Nonetheless, structure-based drug design (SBDD) may be constrained by the accessibility of high-quality 3D structures and the intricacy of the target molecule. Additionally, this necessitates in vitro and in vivo studies to confirm its efficacy and safety [25]. While these limitations can impact the success rate of SBDD and the time required to develop new drugs, advancements in computational biology and experimental techniques are mitigating their effects.

The present study aimed to address this research gap by employing a multi-analytical approach to explore the antioxidant and antibacterial activities of *Blepharis ciliaris* from the Hail Mountains. Additionally, computational modeling will be used to augment the experimental data, providing insights into the molecular processes that underpin these behaviors. This thorough study aims to reveal the potential of *Blepharis ciliaris, therefore* enhancing the understanding of medicinal plant research and emphasizing the underutilized resources of dry and mountainous areas such as Hail.

## 2. Results and Discussion

### 2.1. GC-MS Findings

Methanol was chosen for its effectiveness in extracting polar bioactive compounds, including phenolics and flavonoids, which are crucial for antioxidant and antibacterial functions [26]. The polar nature promotes the dissolution of these compounds, thereby ensuring a comprehensive phytochemical profile. Methanol is commonly utilized in such investigations for its capacity to maintain the stability of polar metabolites, consistent with established practices aimed at optimizing bioactive yield and facilitating precise biological evaluations [27,28].

**Figure 1:**
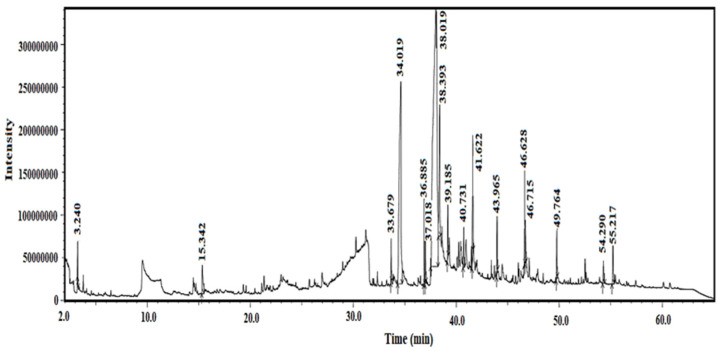
GC-MS chromatogram of the methanol extract of *Blepharis ciliaris*.

The GC-MS chromatogram of the methanol extract from the crude extract of *Blepharis ciliaris*, shown in Figure 1, demonstrates a distinctive composition. This composition was assessed by contrasting several parameters with the National Institute of Standards and Technology Library (NIST) [29], including the mass spectrum’s fragmentation properties, peak retention duration, peak area (%), and aspect ratio. As shown in Table 1, the GC-MS analysis revealed seventeen organic components in the extract. The distinct composition of *Blepharis ciliaris* is highlighted by the discovery that its crude extract contains a range of chemical components in varying amounts.

Rich in fatty acids, each with a distinct percentage composition, the extract offers an intriguing look into its makeup. Notably, the specific percentages of pentadecanoic acid, ethyl methyl ester (2.63%); hexadecanoic acid, methyl ester (1.00%); 9,12-octadecanoic acid (Z, Z)-, methyl ester (2.74%); 9-octadecanoic acid, methyl ester (E) (2.78%); octadecanoic acid (5.88%); 9-tetradecanoic acid (Z) (3.22%); and undec-10-enoic acid, undec-2-n-1-yl ester (5.67%) present a unique and detailed composition of the extract. The high presence of fatty acids in the extract can inhibit the growth of harmful microorganisms, thus increasing its therapeutic efficacy.

The extract, enriched with fatty alcohols such as 7-heptadecyn-1-ol (1.26%) and 41-hexadecyn-1-ol (3.68%), exhibits vigorous biological activity. Notably, these fatty alcohols also exhibit antimicrobial activity, enhancing the extract’s antimicrobial properties and potential therapeutic efficacy. 2-Propanol, 1-[(2-hydroxyethyl)thio]- (1.21%) is an alcohol derivative with a thioether group. The alcohol group is known for its antibacterial properties, which disrupt cell membranes and denature proteins in microorganisms. The inclusion of the sulfur-containing thioether group enhances its biological activity, suggesting that this compound may exhibit increased antibacterial capabilities, thus presenting it as a potential agent for mitigating microbial growth. This extract contains 3.66% piperine, an alkaloid having multifaceted actions, presenting significant promise. Piperine, recognized for its anti-inflammatory, antioxidant, and digestive properties, is also physiologically active. The extract’s antibacterial properties may facilitate the development of effective antimicrobial treatment drugs, making it a promising candidate for future uses.

Additionally, gamma-sitosterol (1.50%) and stigmasterol (2.24%) were shown to reduce cholesterol and improve cardiovascular health. It has been shown that sterols have antibacterial qualities in addition to their therapeutic benefits. These kinds of compounds have been shown to assist the cardiovascular system and aid in the battle against microbial illnesses. In the scientific literature, reports using GC-MS have documented the isolation and identification of steroids and volatile compounds from *Blepharis ciliaris*. El-Shanawany et al. [30] identified a novel compound, stigmasterol tetracosanoate, from the aerial parts of *B. ciliaris*, along with the detection of stigmasterol and stigmasterol-3-O-β-D-glucopyranose in this species. Additionally, Ahmad et al. [31] reported a novel compound, 9-hydroxydodecanoic acid, from the seed oil of another species, *Blepharis scindica*. A separate study on the seeds of *Blepharis persica* employed GC-MS analysis, identifying three predominant compounds: ethyl alpha-D-glucopyranoside (21.05%), 4-vinylphenol (14.5%), and linoleic acid (10.8%) [32]. On the other hand, there is a lack of sufficient data regarding the toxicity levels of Blepharis species, highlighting the need for further research in this area [33].

### 2.2. Antioxidants Activity

In biological systems, free radicals are byproducts of normal metabolic processes. Antioxidants are essential in neutralizing harmful free radicals, thereby providing protection against various diseases and disorders (Ifeanyi, 2018). The antioxidant capacity of *Blepharis ciliaris* was assessed by measuring DPPH, using ascorbic acid as a reference for comparison (Table 2). In biological systems, free radicals are byproducts of normal metabolic processes. Antioxidants are essential in neutralizing harmful free radicals, thereby providing protection against various diseases and disorders [34].

The DPPH assay results for *Blepharis ciliaris* methanol extract demonstrate a concentration-dependent antioxidant activity, with % inhibition increasing from 16.20% at 62.5 µg/mL to 81.55% at 1000 µg/mL. This indicates a robust free-radical-scavenging potential, likely attributable to the bioactive phytochemicals within the extract. The significant differences (*p* < 0.05) compared to the control across all concentrations highlight the extract’s efficacy as a natural antioxidant. These findings position *B. ciliaris* as a promising candidate for developing plant-based antioxidant formulations, warranting further exploration of its phytochemical composition and mechanisms of action. *B. ciliaris*, belonging to the Asteraceae family, has attracted interest due to its significant antioxidant activity. Research indicates that extracts from *B. ciliaris* demonstrates notable free-radical-scavenging capabilities, comparable to other extensively studied plants within the same family [35]. The high total phenolic content of *B. ciliaris* extracts is correlated with their ability as antioxidants. This result is consistent with previous research by Mahboubi et al. [36], who emphasized the effectiveness of other Asteraceae members, including *Taraxacum officinale*, in neutralizing reactive oxygen species. The mechanisms of antioxidant activity in *B. ciliaris* have been investigated through in vitro assays, demonstrating that its bioactive compounds, such as flavonoids and phenolic acids, are essential [37]. *B. ciliaris* remains efficient, especially at greater levels, similar to clove, and has substantial antioxidant activity in comparison to other plant extracts. This is despite the fact that Gulcin et al. [38] discovered that clove had stronger scavenging at lower dosages. There is a considerable inhibition of DPPH by green tea (*Camellia sinensis*), as stated by Chen and Zhang [39]. The inhibition ranges from 60 to 70% at a concentration of 250 µg/mL and can reach up to 85% at a concentration of 1000 µg/mL. Contrarily, the results that *B. ciliaris* produces are akin to those that green tea provides, particularly when the concentrations are higher. When evaluated at a concentration of 1000 µg/mL, the scavenging potential of *B. ciliaris* is practically equal to that of green tea, which is 81.54%. Both *B. ciliaris* and turmeric exhibit comparable levels of DPPH inhibition, with both exhibiting a scavenging activity of 80–85% at a concentration of 1000 µg/mL. This is in accordance with the findings of Menon et al. [40], who discovered zero inhibition rates ranging from 75 to 85% at a concentration of 500–1000 µg/mL. It is probable that the antioxidant capacities of *B. ciliaris* are equivalent to those of other sources. According to Srinivasan [41], fenugreek (*Trigonella foenum-graecum*) exhibits moderate antioxidant activity, blocking 60–65% at 500 µg/mL and up to 75% at 1000 µg/mL. We bring our findings into comparison with the findings of Srinivasan [41]. In contrast, the scavenging performance of *B. ciliaris* is higher, as evidenced by an inhibition of over 81% at a concentration of 1000 µg/mL, in contrast to the 75% inhibition observed for fenugreek. Moreover, according to the DPPH assay, *Blepharis ciliaris* grown in Saudi Arabia demonstrated concentration-dependent scavenging activity by quenching DPPH radicals. The maximum inhibition of DPPH free radicals was observed at 20 mM, with inhibition rates of 88.2%, 87.9%, and 74.2%, respectively [14]. Four phenolic compounds were extracted from the acetone extract of *Blepharis linariifolia*. All compounds had considerable DPPH free-radical-scavenging action, with verbascoside exhibiting the greatest efficacy [16].

### 2.3. Antibacterial Activity

As shown in Table 3, the antimicrobial activity of the methanol extract (1 mg/disk) was statistically compared to ampicillin (10 µg/disk) against the tested bacteria. Ampicillin demonstrated significantly higher inhibition zones than the methanol extract for *S. aureus* and *B. subtilis* (*p* < 0.001), indicating superior activity. For *E. coli*, the difference was significant but less pronounced (*p* = 0.02). Interestingly, the methanol extract showed a significantly higher zone of inhibition against *S. marcescens* compared to ampicillin (*p* < 0.01), highlighting its potential as a natural antimicrobial agent. These findings emphasize the variable efficacy of the methanol extract against different bacterial strains. In the case of *S. marcescens*, a Gram-negative strain frequently resistant to β-lactam antibiotics, ampicillin demonstrated minimal activity (6.00 ± 1.00 mm), whereas the extract showed superior effectiveness (10.00 ± 2.00 mm). The methanolic extract of *B. ciliaris* exhibited notable antibacterial activity, particularly against *B. subtilis*; however, it was less effective than ampicillin, especially against Gram-positive strains. This highlights the potential of natural extracts as adjunct antimicrobial agents. Methanol was selected in this study for extraction because it has been reported as the most effective solvent for extracting a wide range of both polar and non-polar constituents [26]. In the literature, data on the antibacterial activity of *Blepharis ciliaris* are scarce. We found only one article evaluating the antibacterial activity of *Blepharis ciliaris* methanol extracts, showing zones of inhibitions higher than our findings, including 18.5 ± 0.5 mm for *Proteus vulgaris*, 14.5 ± 0.5 for *E. coli*, 14.5 ± 1.5 m for *S. aureus*, and 11.5 ± 0.5 mm for *Bacillus cereus* [42]. The differences may be attributed to variations in the concentrations of the extract used, as we utilized a lower concentration of 1 mg/mL. Worldwide, many studies reported that have antimicrobial activities against bacterial and fungi [30,33,43]. Moreover, seasonal variations influence the phytochemical constituents of plants, which, in turn, affects their antibacterial properties [44]. In Saudi Arabia, a thorough investigation of Saudi Arabian flora in dry regions assessed 24 plant species for their antibacterial efficacy against eight pathogens. Five species—*Echium arabicum*, *Rumex vesicarius*, *Ziziphus nummularia*, *Caylusea hexagyna*, and *Artemisia monosperma*—exhibited considerable antibacterial efficacy against the majority of the evaluated bacteria, yeast fungi [45]. *Acacia gerrardii*, a desert plant native to the Hail region of Saudi Arabia, demonstrated notable antimicrobial activity. The methanolic extract exhibited the highest inhibition zones against methicillin-resistant *Staphylococcus aureus*, *Klebsiella pneumoniae*, *Escherichia coli*, and *Candida* species [46]. Desert plants often exhibit higher antimicrobial activities due to their adaptation to harsh environmental conditions, such as extreme temperatures, limited water availability, and high UV exposure. These stressors drive the production of diverse and unique secondary metabolites, including phenolics, flavonoids, and alkaloids, which serve as defense mechanisms against pathogens and contribute to their antimicrobial properties [47,48].

The MIC test offers a more comprehensive and accurate evaluation of a plant extract’s antibacterial efficacy, augmenting the qualitative results from disk diffusion and promoting the development of plant-derived antimicrobial drugs [49]. The MIC data shown in Table 4 indicate a significant variation in sensitivity to the methanol extract of *Blepharis ciliaris* across the studied microorganisms, with optimal MIC values of 500 µg/mL for both *Staphylococcus aureus* and *Bacillus subtilis*. Moreover, *Escherichia coli* and *Serratia marcescens* had elevated MIC values of 1000 µg/mL for the methanol extract, indicating reduced sensitivity to the extract relative to their MIC values for *Staphylococcus aureus* and *Bacillus subtilis*. The MIC values of the methanolic extract were not comparable to the antibiotic, since the latter is in a pure state, while the extract consists of crude or diverse components. Additionally, as shown in (Table 4), the MIC values reveal a significant disparity in antibacterial potency between the *Blepharis ciliaris* methanol extract and ampicillin. The extract’s MIC was 320-fold higher for *S. aureus* and 160-fold higher for *B. subtilis*, indicating much lower efficacy. For *E. coli* and *S. marcescens*, the fold differences were 40 and 10, respectively. The data suggest that while *Blepharis ciliaris* may exhibit antibacterial capabilities, its effectiveness is much inferior to that of ampicillin, especially against Gram-negative bacteria. This mismatch underscores the need for more research into the active chemicals present in the methanol extract that might augment its antibacterial efficacy and its prospective use in clinical applications. Our findings corroborate the prevailing consensus that Gram-positive bacteria exhibit greater sensitivity to plant extracts compared to Gram-negative bacteria, largely attributable to variations in cell wall architecture [50]. The increased resistance of Gram-negative bacteria is attributed to their complex cell envelope, which includes the inner cytoplasmic membrane, a thin layer of peptidoglycan, and an outer membrane that acts as an additional protective barrier [51]. As in the disk-diffusion test, MIC studies on *Blepharis ciliaris* are scarce. However, it was reported that, the MIC of *Blepharis ciliaris* was >1000 µg/mL for *S. aureus* and *B. subtilis* [52].

More investigations are advised to thoroughly clarify the growth kinetics and mechanisms of action of bacteria subjected to treatment with *Blepharis ciliaris* extract, in both in vitro and in vivo settings. Dirar et al. [43] reported that the majority of pharmacological investigations on *Blepharis* species have been conducted using in vitro systems, with very limited studies utilizing in vivo models. Notably, an in vivo study evaluated the anti-inflammatory activity of *Blepharis maderaspatensis* using Wistar male rats and Swiss albino mice. The ethanolic leaves extract demonstrated significant anti-inflammatory effects, inhibiting paw edema by 84.5% at a dose of 75 mg/kg [53].

### 2.4. Computational Findings

#### 2.4.1. In Silico Identification of Non-Toxic Compounds from *Blepharis ciliaris*

The analysis of *B. ciliaris*-derived compounds conducted via the ProTox 3.0 web server indicated a spectrum of predicted toxicities. Compounds were deemed suitable for biological administration only if their LD50 prediction exceeded Class 3 (toxic if swallowed, 50 < LD50 ≤ 300). The results indicated that compounds 4, 5, and 13 were toxic (LD50 > 5000 mg/kg), whereas compounds 1, 9, 10, and 17 were found to be non-toxic (LD50 < 300 mg/kg) (Table 5), i.e.,; of the 17 compounds, only 3 failed the toxicity prediction analysis. Notably, (+)-Ascorbic acid 2,6-dihexadecanoate, while violating two of Lipinski’s rule of five criteria, is still considered a potential drug candidate. Importantly, to prioritize safety, we calculated the LD50 values for each compound. L-(+)-Ascorbic acid 2,6-dihexadecanoate is a derivative of vitamin C with significant biological activities. It functions as a lipophilic antioxidant and exhibits notable antitumor, wound-healing, and antimicrobial properties [54,55]. Previous in silico studies proposed that (+)-Ascorbic acid 2,6-dihexadecanoate, a compound derived from *Cleome viscosa*, *Acinetobacter baumannii*, and *Parkia timoriana*, may possess inhibitory activities against human papillomavirus, bacterial quorum sensing, and inflammation, respectively [56,57,58].

#### 2.4.2. Drug-Likeness for *Blepharis ciliaris* Compounds

The ADME analysis (absorption, distribution, metabolism, and excretion analysis) enabled the evaluation of the compound’s physical and chemical properties, its potential as a drug, and the identification and removal of phytocompounds lacking significant drug-like characteristics [59]. Lipinski’s rule of five was utilized to evaluate the compounds which exhibited favorable drug-like properties [60]. For instance, molecular weight should be under 500, topological polar surface area must be less than 140 Å^2^, hydrogen bond acceptors should not surpass 5, hydrogen bond donors should also not exceed 5, the octanol–water partition coefficient should be below 5.88, and the number of rotatable bonds should be limited to 10. The in silico predictions generated by the Swiss ADME identified compounds with favorable drug-like characteristics and displayed a range of physicochemical properties (Table 6). The compounds are mostly considered lipophilic (LogP values are mostly positive and indicates lipophilicity), often desirable for oral bioavailability. Furthermore, this molecule exhibited a modest amount of hydrogen bonds, which could affect its solubility and interactions with biological targets. The rule of five (RO5) is a criterion for drug similarity. Compounds that violate the RO5 may encounter difficulties in absorption, distribution, metabolism, and excretion. Based on the data, all chemicals do not breach the RO5.

#### 2.4.3. Structural Characterization of Modeled Proteins

**Figure 2:**
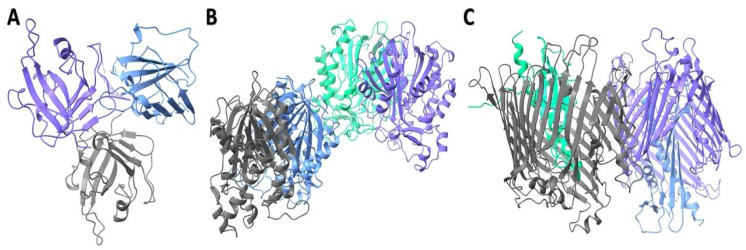
Three-dimensional protein structures. (**A**): *Staphylococcus aureus* sortase A (PDB: 1T2O); (**B**): *Bacillus subtilis* BsFabHB (PDB: 8VDB); (**C**): *E. coli* LPS assembly protein (LptD) (PDB: 4RHB). Uses specific colors to represent the various receptor structures of helices, strands, and *E. coil* with medium state blue, corn flue blue, and dim gray, respectively. Green color represents helices, strands of the particular region.

**Figure 3:**
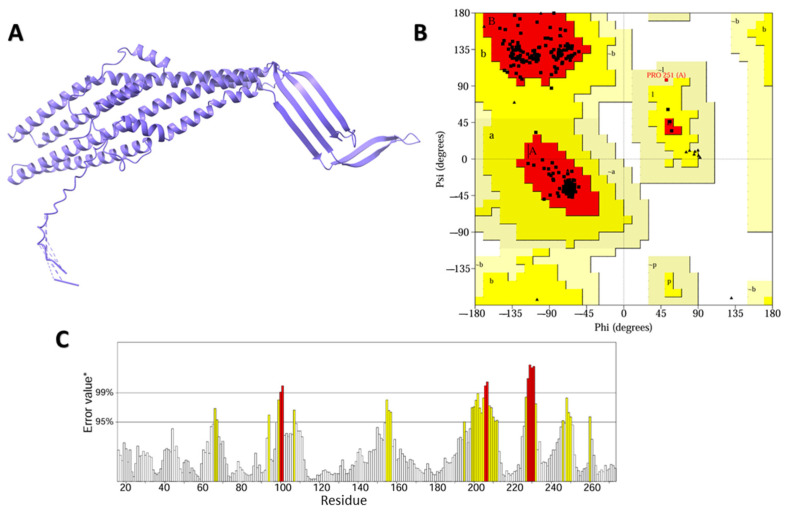
Modeled and structural quality assessment of *Serratia marcescens* outer-membrane protein. (**A**): Outer-membrane protein TolC (**B**): Ramachandran plot analysis, created using SAVES v6.1 web tool; (**C**): TolC protein ERRAT evaluation obtained from the SAVES v6.1 web tool.

Three distinct receptor structures were retrieved from the Protein Data Bank (Figure 2). The model was based on the template B1PN83.1.A, which is the protein EsfC from *Serratia marcescens*. The high sequence identity (96.94%) and coverage (1.00) suggest that EsfC is a close homolog of TolC. The outer-membrane protein TolC structure was predicted using the AlphaFold2 algorithm with high accuracy of global means quantitative error (GMQE) (0.91) (Table S1). In order to verify the quality of the protein structure, we implemented a MolProbity analysis (Table S2). The refinement-improved structural quality of the modeled protein, increased GDT-HA score, decreased RMSD and MolProbity clash score, and reduction in poor rotamers were analyzed (Table S3). The MolProbity analysis revealed 98.8% of the residues in *S. marcescens* outer-membrane protein TolC are in the favored region of the Ramachandran plot, suggesting a high-quality protein structure with minimal conformational issues (Figure 3B and Table S4). Furthermore, the quality factor of 92.7007 for the refined protein suggests a high-quality protein structure according to ERRAT (Figure 3C).

#### 2.4.4. Protein–Ligand Interaction Analysis

**Figure 4:**
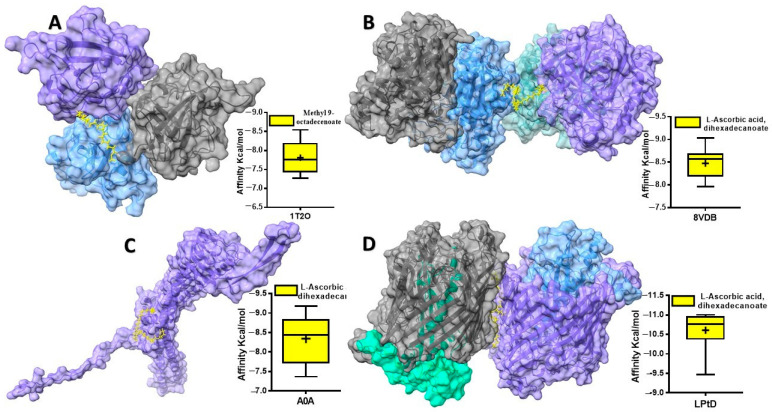
Predicted protein–ligand interaction. The docked compounds are shown in a stick model, colored yellow. (**A**) *S. aureus* sortase A docked with 9-Octadecenoic acid, methyl ester (E); (**B**) *B. subtilis* BsFabHb docked with 3 (+)-Ascorbic acid 2,6-dihexadecanoate; (**C**) *S. marcescens* outer-membrane protein TolC. docked with 3 (+)-Ascorbic acid 2,6-dihexadecanoate; (**D**) *E. coli* LPS assembly protein docked with 3 (+)-Ascorbic acid 2,6-dihexadecanoate. Lower graph represented ten DockThor-VS binding mode scores obtained for the predicted complexes used GraphPad Prism 5 Software.

The outer membrane (in Gram-negative bacteria) and the cell wall (in both Gram-positive and Gram-negative bacteria) act as a protective barrier for the cytoplasmic membrane. For many years, antibiotics have been developed to target the cell wall. However, even if these drugs can pass through the outer membrane, bacteria often have efflux pumps (EPs) that push them back out [7,61]. The binding affinities of 13 compounds derived from *Blepharis ciliaris* with four proteins are presented in Table 7. Binding affinity measures how strongly a compound binds to a protein. A higher negative number predicts the compound binds to the targeted protein more strongly. The binding affinities of the compounds vary significantly across the four proteins. While the 9-Octadecenoic acid, methyl ester (E) compound provided a highest binding affinity docked with *S. aureus* SrtA (Figure 4A), compound 3 (+)-Ascorbic acid 2,6-dihexadecanoate showed a particularly strong affinity for *B. subtilis* BsFabHb, *E. coli* LPS assembly protein, and *Serratia marcescens* outer-membrane protein TolC (Figure 4B–D).

Sortase A (SrtA) is a key enzyme in bacterial cell wall assembly. Its absence leads to impaired surface protein processing and reduced virulence in animal infections. SrtA homologs are widespread among Gram-positive bacteria [62]. Kinetic studies of *S. aureus* sortase A indicate that the enzyme acts as a hydrolase when nucleophilic peptidoglycan or its analogs are absent [63]. *S. aureus* can express up to 21 different proteins on its cell surface [64]. Therefore, targeting SrtA with inhibitor molecules represents a promising approach to deteriorate *S. aureus* virulence and biofilm formation [65]. 9-Octadecenoic acid, also known as oleic acid ester, exhibits anti-inflammatory, antiandrogenic, and anemia-alleviating properties [66,67]. It also exhibits antioxidant activity, antiproliferative and antioxidant, respectively [68,69]. Manilal et al. [70] suggested that the biological activity of *Laurencia brandenii* might be attributed to the presence of octadecadienoic acid, a type of fatty acid. In our study, 9-Octadecenoic acid, methyl ester (E) was found to bind to *S. aureus* SrtA, suggesting a potential role in disrupting the localization of surface proteins to the cell wall envelope [71], thereby hindering its ability to cause infection.

FabH, a key enzyme in fatty acid biosynthesis, plays a crucial role in maintaining bacterial cell membrane integrity [72], by initiating fatty acid synthesis [23] and determining the type of fatty acid chains produced. FabH influences the fluidity and overall properties of the membrane. A *B. subtilis* strain lacking the FabHb gene is susceptible to amycomicin, while a strain lacking the FabHa gene is resistant. Conversely, an *S. aureus* strain overexpressing the *B. subtilis* FabHb gene is resistant to amycomicin, whereas the wild-type *S. aureus* strain is susceptible [73]. *B subtilis* produces branched-chain fatty acids using two FabH isozymes (FabHa and FabHb) [24] for initial condensation and FabF and FabB for subsequent elongation [74]. Plant-derived compounds targeting FabHb could serve as valuable templates for the development of small-molecule FabH inhibitors. Previous research has highlighted fatty acid biosynthesis as a promising target for developing innovative antibacterial drugs [74]. FabH is a promising antimicrobial target, and (+)-Ascorbic acid 2,6-dihexadecanoate has demonstrated its feasibility.

When LPS reaches the inner layer of the outer membrane, the LptDE complex inserts it into the outer layer [75]. While LptD is the only integral outer-membrane protein in the lipopolysaccharide transport (Lpt) pathway and thought to be essential for LPS transport, LptE, is plug within barrel of LptD also considered to as a key for this process [75,76]. Previously, Chng et al. observed an LptD intermediate with slightly faster mobility in *E. coli* with limited LptE [75]. Therefore, downregulating the expression of outer-membrane proteins like LptD could potentially be a promising strategy for combating multidrug-resistant *E. coli* infections. In our study, all *B. ciliaris*-derived compounds demonstrated a highest affinity for the *E. coli* LPS assembly protein, with (+)-Ascorbic acid 2,6-dihexadecanoate exhibiting the strongest binding affinity. Interestingly, L-(+)-Ascorbic acid 2,6-dihexadecanoate, isolated from *Dacryodes edulis*, exhibited potent antibacterial activity against *S. aureus*, *E. coli*, *Streptococcus pneumoniae*, and *Proteus mirabilis* [77].

*Serratia marcescens*, a soil-dwelling bacterium, is a significant opportunistic pathogen, particularly threatening immunocompromised individuals [78,79]. Its high intrinsic antibiotic resistance [80], including resistance to tigecycline and other major antibiotic classes [81], makes treatment of infections challenging. Gram-negative bacteria often employ nodulation division (RND)-type efflux systems, which typically consist of three components: a cytoplasmic membrane pump, a periplasmic component, and an outer-membrane channel [82]. However, the specific outer-membrane channels involved in *S. marcescens* are not well characterized. TolC promotes the evacuation of substrates from cells. The TolC homologue, EsfC, is implicated in efflux and is closely related to TolC in structure. Interestingly, we demonstrated that (+)-Ascorbic acid 2,6-dihexadecanoate exhibits favorable binding interactions that may interfere with TolC-mediated efflux and potentially weaken the bacterial cell wall. Further in vitro studies are necessary to validate these findings.

Finally, it is important to mention that the possible utilization of *B. ciliaris*-derived compounds from Saudi Arabia has both economic and ecological implications. Economically, these bioactive compounds offer potential for developing cost-effective, plant-based pharmaceuticals, nutraceuticals, and cosmetics, reducing reliance on synthetic alternatives. This can stimulate local industries, create job opportunities, and add value to underutilized desert flora. Ecologically, promoting the use of native plants like *B. ciliaris* supports biodiversity conservation and sustainable resource management. However, large-scale harvesting must be carefully regulated to prevent overexploitation and habitat degradation, ensuring long-term ecological balance while leveraging its economic benefits.

## 3. Materials and Methods

### 3.1. Chemicals

All chemicals used in this study were of analytical grade. The chemicals and indicators were utilized as received, without any purification, and were procured from Merck (Merck KgaA, Darmstadt, Germany). Bacteriological media were obtained from Oxoid Ltd., UK. Folin–Ciocalteu’s phenol reagent, standard vitamin E, and DPPH (2,2-Diphenyl-1-picrylhydrazyl) reagents, along with other chemicals, such as aluminum chloride hexahydrate, quercetin, and H_2_SO_4_, were sourced from Sigma-Aldrich (St. Louis, MI, USA).

### 3.2. Plant Samples and Extraction

**Figure 5:**
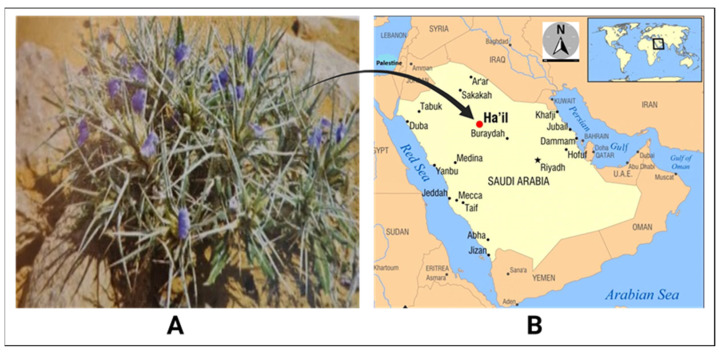
The plant sample and the collection site: (**A**) *Blepharis ciliaris*; (**B**) the location of collection, Ha’il area.

The aerial parts of *Blepharis ciliaris* (L.) B.L. Burtt were collected from the Hail region in northern central Saudi Arabia (Figure 5), where the plant grows on mountainous slopes (27°25′9.040″ N, 41°25′9.330″ E) between March and May 2023. The plant species was authenticated by a botanist from the Biology Department at Hail University. The harvested plant material was cleaned, air-dried in the shade, and ground into a fine powder. One hundred grams of the powdered plant material was macerated in 1000 mL of 80% methanol for three days at ambient temperature (approximately 35–37 °C) with regular agitation. The methanol was then evaporated, and the extract was concentrated using a rotary vacuum evaporator (8 kW, 50 L, Henan Lanphan Industry Co., Ltd., Zhengzhou, China). The methanolic extract was utilized in antibacterial and antioxidant evaluations, while a portion of the crude methanol extract was diluted in hexane, microfiltered, and injected into the GC–MS for analysis.

### 3.3. Bacterial Samples

Bacterial strains, including methicillin-resistant *Staphylococcus aureus* (MRSA), *Bacillus subtilis*, *Escherichia coli*, and *Serratia marcescens*, were generously supplied by King Khalid Hospital in Hail. Upon receipt, the strains were maintained on a brain heart infusion (BHI) agar medium at 4 °C and subsequently re-cultured prior to use in further analyses.

### 3.4. GC-MS Analysis

#### 3.4.1. Test Conditions

A split ratio of 10:1 was used for the 1 µL sample injection. The oven temperature was set at 60 °C, rising by 80 °C every minute until it reached 280 °C, which was maintained for 25 min, culminating in a total duration of 53.5 min. The GC-MS analysis of the leaf oil identified fatty acid methyl esters (FAMEs) at the specified conditions: Helium served as the carrier gas at a flow rate of 0.7 mL/min, with the ion source, transfer line, and injector temperatures maintained at 250 °C, 250 °C, and 220 °C, respectively. The oven temperature was originally set at 50 °C for 8 min and then increased to 250 °C at a rate of 40 °C per minute. Data collection included capturing full-scan mass spectra throughout a range of 35–500 amu. Unidentified chemicals were recognized by comparing the obtained spectra with recognized mass spectral libraries. Calibration and detection limits were established in accordance with the manufacturer’s requirements, with additional information available in another source [83,84].

#### 3.4.2. The Experiment of GC-MS

A gas chromatography–mass spectrometry (GC–MS) analysis was conducted utilizing a Perkin Elmer Clarus 600 GC system (Perkin Elmer, Waltham, MA, USA), equipped with a Rtx 5MS capillary column (30 m length, 0.25 mm internal diameter, 0.25 µm film thickness, maximum temperature 350 °C) and connected to a Perkin Elmer Clarus 600C mass spectrometer. Ultra-high-purity helium (99.9999%) functioned as the carrier gas at a steady flow rate of 1.0 mL/min. The temperatures of the ion source, transfer line, and injector were sustained at 280 °C, 270 °C, and 270 °C, respectively. The device functioned at ionization energy of 70 eV, and the electron multiplier (EM) voltage was established by auto-tuning. Mass spectral data were obtained in full-scan mode, including a mass range of 40 to 550 amu [85,86].

### 3.5. DPPH Radical Scavenging

The antioxidant capacity of *Blepharis ciliaris* was evaluated through the DPPH radical scavenging assay, following a previously established protocol [87]. Methanolic solutions of varying extract concentrations were prepared alongside a 0.1 mM DPPH solution. The mixture’s absorbance was recorded via UV–visible spectrophotometry. A control solution was also prepared, and the percentage of DPPH radical scavenging activity was determined using the formula:

*Inhibition* (%) = (*Ac* − *As*)/*Ac* × 100

where “Ac” represents the absorbance of the control, and “As” represents the absorbance in the presence of the plant extract or standard. A plot of extract concentration (lg extract/mL of DPPH solution) versus the percentage inhibition enabled the calculation of the half-maximal inhibitory concentration (IC_50_), representing the concentration (mg/mL) required to achieve 50% radical scavenging, derived through interpolation.

### 3.6. Disc Diffusion Test

The antibacterial potential of *Blepharis ciliaris* was determined using the disk-diffusion technique. Bacterial suspensions of MRSA, *Bacillus subtilis*, *Escherichia coli*, and *Serratia marcescens* were adjusted to the McFarland standard, yielding approximately 10^6^ CFU/mL, and uniformly spread on sterile Mueller–Hinton agar plates (20 mL per plate). Sterile filter disks (6 mm diameter, Whatman No. 1) were impregnated with 1 mg/disk of methanolic extract diluted in 10% DMSO (Dimethyl sulfoxide). A volume of 10 µL of the 100 mg/mL methanolic extract solution was absorbed by the paper disk, containing 1 mg/disk of the extract; 80% methanol has no effect on bacterial growth. The saturated disks were placed on the inoculated agar under aseptic conditions. The plates were incubated in an incubator at 35 °C for 24 h. Disks treated with 10% DMSO served as the negative control, while ampicillin (10 μg/disk) was used as the positive control. After incubation, the halo around the disks were measured to the nearest millimeters (mm) and results were averaged from three independent experiments [88].

### 3.7. Determination of MIC

The minimum inhibitory concentration (MIC) of *Blepharis ciliaris* methanolic extract was determined using a microdilution method in 96-well microplates. Serial two-fold dilutions of the extract (ranging from 1000 to 15.6 µg/mL) were prepared in 10% DMSO across the microplate rows. Also, ampicillin was serially diluted in a grange from 100 to 1.56 µg/mL, in a separate raw at the 96-well microplate to serve as positive control. A total of 20 µL of bacterial suspensions, standardized to 0.5 McFarland, and 160 µL of Mueller–Hinton broth were added to each well. The microplates were incubated at 37 °C for 24 h. After incubation, 40 µL of 2,3,5-triphenyltetrazolium chloride (TTC) at a concentration of 0.2 g/mL was added to each well and incubated for 30 min at 37 °C. TTC acts as an indicator of bacterial growth by staining live bacterial cells with a red dye. The MIC was defined as the lowest concentration of extract in wells where no visible bacterial growth was observed [89].

### 3.8. Toxicity Prediction of Blepharis Ciliaris-Derived Compounds

To assess the potential safety of *Blepharis ciliaris*-derived compounds, in silico prediction web server of ProTox 3.0 (https://tox.charite.de/protox3/) was used (accessed on 7 October 2024). This website has a gathering of computer models that can predict different types of toxicity for a compound for each endpoint [90]. The compounds were converted to SMILE format using Avogadro version 1.2.0 software (Table S5).

### 3.9. Physicochemical Properties for Blepharis ciliaris Compounds

We assessed drug-likeness for the selected compounds. We utilized the freely available SwissADME web tool (www.swissadme.ch) (accessed on 21 October 2024), developed by the Swiss Institute of Bioinformatics [91]. The web tools offer a computational approach (in silico) to predict various pharmacokinetic (ADME) properties of the shortlisted compounds, including absorption, distribution, metabolism, and excretion. The SDF files were uploaded into the SwissADME web (accessed on 8 October 2024).

### 3.10. Retrieval of Targeted Receptor Proteins

Three receptor structures of *Staphylococcus aureus* sortase A (PDB: 1T2O), *Bacillus subtilis* BsFabHb (PDB: 8VDB), and *E. coli* LPS assembly protein (LptD) (PDB: 4RHB) (Figure 2) were retrieved from the Protein Data Bank (PDB) (https://www.rcsb.org/) (accessed on 15 October 2024). Since the *Serratia marcescens* outer-membrane protein TolC crystal structure was unavailable in the PDB, the sequences were retrieved from (https://www.uniprot.org/) (UniProt ID: A0A1C3HBD9) (accessed on 15 October 2024), and the sequences were saved in FASTA format. A protein model for the *Serratia marcescens* was constructed using template-based modeling with Swiss-model (https://swissmodel.expasy.org/) (accessed on 15 October 2024).

### 3.11. Proteins Refinement and Validation for S. marcescens

To enhance the protein prediction profile, the generated protein structures were refined using ModRefiner [92]. A Ramachandran plot analysis then evaluated the refined structures compared to the originals. ERRAT and PROCHECK tools (https://bioserv.rpbs.univ-paris-diderot.fr/services/PEP-FOLD/) (accessed on 15 October 2024) were used to validate the refined predicted structure, using SAVES v6.1 web tool (https://saves.mbi.ucla.edu/) (accessed on 16 October 2024).

### 3.12. Ligand Preparation

Non-toxic *Blepharis ciliaris*-derived compounds were selected for molecular docking (Table S1). Ligands were retrieved from the PubChem-NCBI database (https://pubchem.ncbi.nlm.nih.gov/) (accessed on 14 October 2024) in structure data file (SDF) format.

### 3.13. Protein–Ligand Docking

For molecular docking prediction, proteins and ligands were uploaded to DockThor-VS web server (https://dockthor.lncc.br/v2/) (accessed on 17 October 2024). DockThor-VS offer a multi-step protein–ligand docking process. First, protein and ligand files were uploaded, followed by prepares the protein by adding missing hydrogen atoms, completing side chains, and changing protonation states. Then, the molecules were prepared by adding hydrogen atoms, freezing rotatable bonds, and obtaining MMFF94S atom types and partial charges. Next, the binding site was defined with used the blind docking option. Finally, the protein was docked to the ligand using a genetic algorithm [92]. The number of binding modes and the predicted binding affinity were calculated. UCSF ChimeraX version 1.8 was used to visualize the predicted protein–ligand complexes [93]. GraphPad Prism 5 Software (San Diego, CA, USA) generated graphs for the top 10 runs.

### 3.14. Statistical Analysis

The tests were conducted in triplicates. Results are presented as mean ± standard deviation. Statistical analysis was conducted with GraphPad Prism software (version 10.0).

## 4. Conclusions

*Blepharis ciliaris*, sourced from the Hail Mountains in Saudi Arabia, demonstrates noticeable antioxidant and antibacterial potential. GC-MS analysis identified 17 bioactive compounds, while DPPH assays confirmed notable antioxidant activity. The plant exhibited varying degrees of antibacterial efficacy against both Gram-positive and Gram-negative bacteria. Computational simulations revealed key interactions between specific phytochemicals and bacterial target proteins, suggesting that isolating and testing these compounds could enhance their antibacterial potential. Future studies should focus on the pharmacokinetics and evaluating the antibacterial and antioxidant properties of *Blepharis ciliaris* using advanced in vivo models. Expanding computational investigations to additional bacterial targets and exploring structural analogs of its active compounds may pave the way for the development of novel plant-based antibacterial agents. Furthermore, studies on bioavailability and pharmacokinetics are crucial to optimize these compounds for therapeutic applications. The potential integration of *Blepharis ciliaris* extracts with conventional antibiotics to combat multidrug-resistant infections represents a significant avenue for addressing global public health challenges.

## Figures and Tables

**Table 1:** Chemical compounds in the methanolic extract of *Blepharis ciliaris* analyzed using GC-MS.

Peak	Compound Name	Area%	Error%	Ret. Time (min)	Classification
1	2-Propanol, 1-[(2-hydroxyethyl)thio]-	1.21	±0.05	3.240	Alcohol
2	Pentadecanoic acid, ethyl methyl ester	2.63	±0.06	15.342	Fatty Acid
3	Hexadecanoic acid, methyl ester	1.00	±0.04	33.679	Fatty Acid
4	(+)-Ascorbic acid 2,6-dihexadecanoate	15.63	±0.10	34.620	Vitamin Compound
5	9,12-Octadecadienoic acid (Z,Z)-, methyl ester	2.74	±0.03	36.885	Fatty Acid
6	9-Octadecenoic acid, methyl ester (E)	2.78	±0.03	37.018	Fatty Acid
7	7-Heptadecyn-1-ol	1.26	±0.05	38.393	Fatty Alcohol
8	Octadecanoic acid	5.88	±0.07	39.185	Fatty Acid
9	9-Octadecanoic acid (Z), 2,3-dihydro-	2.94	±0.02	40.731	Fatty Acid
10	7-Heptadecenoic acid	1.90	±0.02	41.622	Fatty Acid
11	41-Hexadecyn-1-ol	3.68	±0.04	46.628	Fatty Alcohol
12	Undec-10-ynoic acid, undec-2-en-1-yl ester	5.67	±0.08	46.680	Fatty Acid
13	9,12-Octadecadienoic acid (Z,Z)-, methyl ester	2.78	±0.03	46.868	Fatty Acid
14	Piperine	3.66	±0.06	49.764	Alkaloid
15	Stigmasterol	2.24	±0.02	54.290	Sterol
16	gamma-Sitosterol	1.50	±0.01	55.217	Sterol
17	9-Tetradecadenoic acid (Z)	3.22	±0.05	55.511	Fatty Acid

**Table 2:** Antioxidant assay DPPH of *B. ciliaris*.

Concentration of *B. ciliaris* (µg/mL)	1	2	3	Mean	SD	% Inhibition	*p*-Value *	Significance (vs. Control)
62.5	0.364	0.386	0.367	0.3723	0.0119	16.20	<0.05	Significant
125	0.264	0.286	0.267	0.2723	0.0119	38.71	<0.01	Highly Significant
250	0.19	0.198	0.189	0.1923	0.0049	56.71	<0.01	Highly Significant
500	0.185	0.184	0.145	0.1713	0.0208	61.44	<0.001	Highly Significant
1000	0.11	0.12	0.016	0.0820	0.0574	81.55	<0.001	Highly Significant
Control	0.42	0.449	0.464	0.4443	0.0224	0.00	-	-

* *p*-values were calculated using one-way ANOVA followed by Tukey’s post hoc test (or an equivalent test) for pairwise comparisons between the control and each concentration. SD: standard deviation.

**Table 3:** Antibacterial activity of methanol extract of *Blepharis ciliaris* and ampicillin (mean zone of inhibition ± SD).

Bacteria	Methanol Extract (1 mg/disk)	Ampicillin (10 µg/disk)	*p*-Value	Significance (Methanol vs. Ampicillin)
*S. aureus*	10.33 ± 1.53	25.0 ± 2.0	<0.001	Highly Significant
*B. subtilis*	13.33 ± 1.53	23.0 ± 2.5	<0.001	Highly Significant
*E. coli*	10.67 ± 1.53	12.0 ± 1.5	0.02	Significant
*S. marcescens*	10.00 ± 2.00	6.0 ± 1.0	<0.01	Highly Significant

**Table 4:** The MIC ratios of *Blepharis ciliaris* methanol extract (80% *v*/*v*) against the tested bacteria.

Bacteria	MIC Ratios in (µg/mL)		Fold Difference (Extract/Ampicillin)
	Methanol Extract MIC (µg/mL)	Ampicillin MIC (µg/mL)	
*S. aureus*	500	1.56	320
*B. subtilis*	500	3.125	160
*E. coli*	1000	25	40
*S. marcescens*	1000	100	10

**Table 5:** Predicted oral toxicity of *Blepharis ciliaris* compounds from ProTox-II web server.

No.	Compound Name	LD50 (mg/kg)	Predicted Toxicity Class *	Average Similarity (%)	Prediction Accuracy (%)
1	2-Propanol, 1-[(2-hydroxyethyl)thio]-	300	3	59.57	67.38
2	Pentadecanoic acid, ethyl methyl ester	5000	5	100	100
3	Hexadecanoic acid, methyl ester	5000	5	100	100
4	(+)-Ascorbic acid 2,6-dihexadecanoate	10,000	6	85.78	70.97
5	9,12-Octadecadienoic acid (Z,Z)-, methyl ester	20,000	6	85.93	70.97
6	9-Octadecenoic acid, methyl ester (E)	3000	5	89.64	70.97
7	7-Heptadecyn-1-ol	753	4	67.58	68.07
8	Octadecanoic acid	900	4	100	100
9	9-Octadecanoic acid (Z), 2,3-dihydro-	48	2	100	100
10	7-Heptadecenoic acid	48	2	100	100
11	41-Hexadecyn-1-ol	520	4	54.95	67.38
12	Undec-10-ynoic acid, undec-2-en-1-yl ester	5000	5	81.82	70.97
13	9,12-Octadecadienoic acid (Z,Z)-, methyl ester	20,000	6	85.93	70.97
14	Piperine	500	4	53.02	67.38
15	Stigmasterol	890	4	89.38	70.97
16	gamma-Sitosterol	890	4	89.38	70.97
17	9-Tetradecadenoic acid (Z)	48	2	100	100

* Class 1: fatal if swallowed (LD50 ≤ 5); Class 2: fatal if swallowed (5 < LD50 ≤ 50); Class 3: toxic if swallowed (50 < LD50 ≤ 300); Class 4: harmful if swallowed (300 < LD50 ≤ 2000); Class 5: may be harmful if swallowed (2000 < LD50 ≤ 5000); Class 6: nontoxic (LD50 > 5000).

**Table 6:** Physicochemical properties and drug-likeness of *Blepharis ciliaris* compounds.

Compound Name	Molecular Weight	Hydrogen Bonds		Log P * (iLogPo/w)	Molar Refractivity	RO5 Violation **
		Acceptor Donor				
2-Propanol, 1-[(2-hydroxyethyl)thio]-	120.21	1	0	2.08	35.16	0
Pentadecanoic acid, ethyl methyl ester	270.45	2	0	4.67	85.12	1
Hexadecanoic acid, methyl ester	270.45	2	0	4.41	85.12	1
(+)-Ascorbic acid 2,6-dihexadecanoate	654.96	8	2	6.91	188.84	2
9,12-Octadecadienoic acid (Z,Z)-, methyl ester	294.47	2	0	4.43	93.78	1
9-Octadecenoic acid, methyl ester (E)	310.51	2	0	5.24	99.06	1
7-Heptadecyn-1-ol	238.41	1	1	4.23	78.35	1
Octadecanoic acid	284.48	2	1	4.3	90.41	1
9-Octadecanoic acid (Z), 2,3-dihydro-	296.49	2	1	3.68	94.74	1
7-Heptadecenoic acid	268.43	2	1	3.9	85.13	1
Hexadecynol	238.41	1	1	4.3	78.07	1
Undec-10-ynoic acid, undec-2-en-1-yl ester	320.51	2	0	5.25	102.03	1
9,12-Octadecadienoic acid (Z,Z)-, methyl ester	294.47	2	0	4.43	93.78	1
Piperine	265.26	3	1	2.3	77.35	0
Stigmasterol	412.69	1	1	5.08	132.75	1
gamma-Sitosterol	414.71	1	1	5.05	133.23	1
9-Tetradecadenoic acid (Z)	226.36	2	1	3.39	70.71	0

*: octanol–water partition coefficient; **: Lipinski rule of five.

**Table 7:** Binding affinity (kcal/mol) of *Blepharis ciliaris*-derived compounds.

No.	Compound Name	1T2O *	8VDB **	4RHB ***	A0A ****
1	Pentadecanoic acid, ethyl methyl ester	−5.979	−6.696	−6.112	−6.194
2	Hexadecanoic acid, methyl ester	−7.469	−7.818	−7.842	−7.612
3	(+)-Ascorbic acid 2,6-dihexadecanoate	−7.838	−8.237	−10.768	−9.063
4	9,12-Octadecadienoic acid (Z,Z)-, methyl ester	−7.67	−7.672	−8.939	−7.776
5	9-Octadecenoic acid, methyl ester (E)	−8.543	−7.927	−8.945	−7.839
6	7-Heptadecyn-1-ol	−7.358	−7.326	−8.277	−7.626
7	Octadecanoic acid	−7.201	−7.599	−8.124	−6.791
8	Hexadecynol	−7.079	−7.24	−8.381	−7.461
9	Undec-10-ynoic acid, undec-2-en-1-yl ester	−7.505	−7.282	−9.38	−7.982
10	9,12-Octadecadienoic acid (Z,Z)-, methyl ester	−7.67	−7.672	−8.939	−7.776
11	Piperine	−8.05	−7.22	−8.547	−7.496
12	Stigmasterol	−8.316	−7.094	−9.77	−7.949
13	gamma-Sitosterol	−8.276	−7.3	−9.525	−8.131

* *Staphylococcus aureus* sortase A; ** *Bacillus subtilis* BsFabHB; *** *E. coli* LPS assembly protein (LptD); **** *Serratia marcescens* outer-membrane protein TolC.

## Data Availability

Data are contained within the article.

## Supplementary Materials

The following supporting information can be downloaded at https://www.mdpi.com/article/10.3390/plants13243491/s1: Table S1: Structural characterization of *S. marcescens* outer-membrane protein TolC using AlphaFold2; Table S2: Structural quality assessment of *S. marcescens* outer-membrane protein TolC using MolProbity; Table S3: Refinement on structural quality of *S. marcescens* outer-membrane protein TolC; Table S4: Verified structural quality of *S. marcescens* outer-membrane protein TolC; Table S5: Simplified Molecular Input Line Entry System (SMILES) and Chemical Identifier (CID) for *Blepharis ciliaris*-derived compounds.
