# Supplementary material for: Comprehensive In Vitro Evaluation of Antibacterial, Antioxidant, and Computational Insights into Blepharis ciliaris (L.) B. L. Burtt from Hail Mountains, Saudi Arabia
Source: Plants (Basel). 2024 Dec 13;13(24):3491. doi: 10.3390/plants13243491 (PMC11728784; doi:10.3390/plants13243491)
Supplement: Supplementary file 1 [file plants-13-03491-s001.zip › plants-3370445-supplementary.pdf]

plants

MDPI

Supplementary materials

Table S1. Structural characterization of *S. marcescens* outer membrane protein TolC using AlphaFold2

Feature	Value
Template	B1PN83.1.A (EsfC)
AlphaFold DB model	B1PN83_SERMA (gene: esfC, organism: *Serratia marcescens*)
Biounit oligo state	Monomer
Method	AlphaFold v2
Sequence similarity	0.59
Coverage	1.00
Range	1-458
Biounit	Monomer
QSQE	N/A
Oligo State	N/A

Table S2. Structural quality assessment of *S. marcescens* outer membrane protein TolC using MolProbity

Category	Value	Details
MolProbity Score	0.89	Overall quality of the structure
Clash Score	0	Number of steric clashes
Ramachandran Favored	96.93%	Percentage of residues in the favored region of the Ramachandran plot
Ramachandran Outliers	0.88%	Percentage of residues in outlier regions of the Ramachandran plot
Ramachandran Outlier Residues	A2, A6, A7, A3	Residues in outlier regions
Rotamer Outliers	1.86%	Percentage of residues with unfavorable sidechain conformations
Rotamer Outlier Residues	A18, A27, A416, A11, A1, A17, A15	Residues with unfavorable sidechain conformations
C-Beta Deviations	3	Number of residues with large C-beta deviations
C-Beta Deviation Residues	A2, A371, A8	Residues with large C-beta deviations
Bad Bonds	1/3619	Number of bad bonds
Bad Bond Residue	A257 TRP	Residue with a bad bond
Bad Angles	12/4902	Number of bad angles
Bad Angle Residues	A40, A446, A285, A432, (A306 SER-A307 ASP), A190, A8, A90, A454, (A4 SER-A5 LYS), (A325 LEU-A326 PRO), A3 GLN	Residues with bad angles
Cis Prolines	1/13	Number of cis proline residues
Cis Proline Residue	(A250 ILE-A251 PRO)	Cis proline residue
Twisted Non-Prolines	4/444	Number of twisted non-proline residues
Twisted Non-Proline Residues	(A2 ILE-A3 GLN), (A5 LYS-A6 ARG), (A6 ARG-A7 GLN), (A7 GLN-A8 ALA)	Twisted non-proline residues

Table S3. Refinement on structural quality of *S. marcescens* outer membrane protein TolC

Model	GDT-HA	RMSD	MolProbity	Clash Score	Poor Rotamers	Rama Favored
Initial	1	0	1.126	1	1.6	96.9
MODEL 1	0.982	0.323	1.352	6.4	0.5	99.3
MODEL 2	0.9787	0.323	1.336	6.1	0.8	99.3
MODEL 3	0.9705	0.352	1.284	5.3	0.5	99.3
MODEL 4	0.9798	0.322	1.322	5.6	1.1	99.1
MODEL 5	0.9749	0.356	1.345	6.3	0.8	99.6

Table S4. Verified structural quality of *S. marcescens* outer membrane protein TolC

**Category**	**Value**
Ramachandran Plot	98.8% core, 1.2% allowed, 0.0% general, 0.0% disallowed
Labelled Residues	3 (out of 456)
Chi1-chi2 Plots	0 labelled residues (out of 266)
Side-chain Params	5 better, 0 inside, 0 worse
Max. Deviation	10.5
Bad Contacts	1
Bond len/angle	7.1
Morris et al Class	1 1 2001
G-factors	Dihedrals: 0.60, Covalent: -0.14, Overall: 0.31
Planar Groups	100.0% within limits, 0.0% highlighted

Table S5. Simplified Molecular Input Line Entry System (SMILES) and **Chemical Identifier (CID) for** *Blepharis ciliaris*-derived compounds.

No	**Compound name**	**SMILES**	**CID**
1	Pentadecanoic acid, ethyl methyl ester	CCCCCCCCCCCCCCC(=O)OCC	23518
2	Hexadecanoic acid, methyl ester	CCCCCCCCCCCCCCCC(=O)OC	8191
3	(+)-Ascorbic acid 2,6-dihexadecanoate	CCCCCCCCCCCCCCCCC(=O)OC(CCCCCCCCCCC(=O)OCC1OC(=O)C(C1O)O)CCCC	168814
4	9,12-Octadecadienoic acid (Z,Z)-, methyl ester	CCCCCCCCC/C=C/C/C=C/CCCC(=O)OC	14122955
5	9-Octadecenoic acid, methyl ester (E)	CCCCCCCCC/C=C/CCCCCCCC(=O)OC	8202
6	7-Heptadecyn-1-ol	CCCCCCCCCCC#CCCCCO	5364485
7	Octadecanoic acid	CCCCCCCCCCCCCCCCCC(=O)O	5282750
8	Hexadecynol	CCCCCCCCCCCCCCC#CO	22348650
9	Undec-10-ynoic acid, undec-2-en-1-yl ester	C=CCCCCCCCCCOC(=O)CCCCCCCC#C	129858038
10	9,12-Octadecadienoic acid (Z,Z)-, methyl ester	CCCCCCCCC/C=C/C/C=C/CCCC(=O)OC	3931
11	Piperine	O=c1ccc2c(c(=O)[nH]1)cccc2Oc1ccccc1	638024
12	Stigmasterol	CC[C@@H](C(C)C)/C=C/[C@H]([C@H]1CC[C@@H]2[C@]1(C)CC[C@H]1[C@H]2CC=C2[C@]1(C)CC[C@@H](C2)O)C	5280794
13	gamma-Sitosterol	CC[C@@H](C(C)C)CC[C@H]([C@H]1CC[C@@H]2[C@]1(C)CC[C@H]1[C@H]2CC=C2[C@]1(C)CC[C@@H](C2)O)C	222284
